# Interaction of the Mechano-Electrical Feedback With Passive Mechanical Models on a 3D Rat Left Ventricle: A Computational Study

**DOI:** 10.3389/fphys.2019.01041

**Published:** 2019-09-24

**Authors:** Minh Tuấn Du'o'ng, David Holz, Muhannad Alkassar, Sven Dittrich, Sigrid Leyendecker

**Affiliations:** ^1^Chair of Applied Dynamics, University of Erlangen-Nuremberg, Erlangen, Germany; ^2^School of Mechanical Engineering, Hanoi University of Science and Technology, Hanoi, Vietnam; ^3^Pediatric Cardiology, University of Erlangen-Nuremberg, Erlangen, Germany

**Keywords:** electromechanics, mechano-electrical feedback, passive mechanics, compressible, incompressible, exponential, polynomial, rat left ventricle

## Abstract

In this paper, we are investigating the interaction between different passive material models and the mechano-electrical feedback (MEF) in cardiac modeling. Various types of passive mechanical laws (nearly incompressible/compressible, polynomial/exponential-type, transversally isotropic/orthotropic material models) are integrated in a fully coupled electromechanical model in order to study their specific influence on the overall MEF behavior. Our computational model is based on a three-dimensional (3D) geometry of a healthy rat left ventricle reconstructed from magnetic resonance imaging (MRI). The electromechanically coupled problem is solved using a fully implicit finite element-based approach. The effects of different passive material models on the MEF are studied with the help of numerical examples. It turns out that there is a significant difference between the behavior of the MEF for compressible and incompressible material models. Numerical results for the incompressible models exhibit that a change in the electrophysiology can be observed such that the transmembrane potential (TP) is unable to reach the resting state in the repolarization phase, and this leads to non-zero relaxation deformations. The most significant and strongest effects of the MEF on the rat cardiac muscle response are observed for the exponential passive material law.

## 1. Introduction

Cardiovascular diseases remain the leading causes of death, e.g., 30% in the US and 45% in Europe (Wilkins et al., 2017), even though the cardiovascular system has been extensively studied. A great hope to reduce the mortality of cardiovascular diseases in the future lies in computational models. These models can be an effective tool to study and understand the cardiovascular system and related pathologies in a new fashion. This includes, e.g., the early recognition of heart failure, better understanding of the cardiac function under normal and pathological conditions (Trayanova, 2011; Gao et al., 2015), patient-specific diagnostics and treatments (Asner et al., 2017), as well as the development of cardiac devices (Baillargeon et al., 2015).

Computational models of the heart include an excitation-contraction coupling representing the physiological course of converting an electrical stimulus into an active muscle contraction. The electrical excitation, which induces depolarization and contraction of cardiac cells, is widely represented by the ionic Hodgkin-Huxley model for neurons (Hodgkin and Huxley, 1952). Based on the Hodgkin-Huxley description, a large number of models have been derived, which can be subdivided into physiological and phenomenological models. On the one hand, physiological models are used from cell to tissue level, for example drug testing of a cell drum compromising three different cardiac stem cells (Frotscher et al., 2016) as well as for drug investigations on a complete human heart (Costabal et al., 2018). On the other hand, phenomenological approaches like the Aliev-Panfilov model and the FitzHugh-Nagumo model are also widely used to model cardiac excitability, mostly at tissue level (Fitzhugh, 1961; Rogers and McCulloch, 1994; Aliev and Panfilov, 1996). Compared to physiological models, the phenomenological approach describes the TP evolution, using a significantly smaller number of internal variables, while still capturing the main characteristics of the cardiac electrophysiology. Thus, it is less complex, easier to implement, reduces computational costs (Cherubini et al., 2008) and is often used to investigate arrhythmia.

Apart from the excitation-induced contraction of cardiac cells, the depolarization and subsequently the contraction of cardiomyocytes can be also initiated through the stretch-induced opening of ion channels, commonly referred to as the mechano-electrical feedback (MEF) (Kohl et al., 1999; Keldermann et al., 2009). More specifically, the MEF is capable of modifying the electrophysiology (Kamkin et al., 2005) and might promote stretch-activated arrhythmias, which have been commonly identified as a result of electrical disorders in the cardiac muscle (Franz, 1996). Moreover, the MEF can shorten the action potential (AP) duration or shorten the time course of repolarization (Franz, 1996) and break up spiral waves (Panfilov et al., 2007). Furthermore, it has been shown that high strain and stretch rates can cause premature ventricular excitation (Franz et al., 1992; Hu and Sachs, 1997). In addition to the extensive investigations on humans, similar findings have been observed for arterial cardiomyocytes in rats (Kamkin et al., 2000). In particular, stretch can result in atrial fibrillation after ventricular infarction (Kamkin et al., 2000). Also in other mammals like rabbits, the MEF can give rise to spontaneous arrhythmia in an acute local ischemia (Jie et al., 2010). This phenomenon plays a key role in interpreting the interplay between the electrophysiology and mechanics of cardiomyocytes (Keldermann et al., 2007). In a comprehensive computational modeling approach for cardiac electromechanics, it is essential to account not only for the excitation-triggered contraction of cardiac myocytes but also for the stretch-activated excitation. To investigate the effect of the MEF, strongly coupled electromechanical models are used (Costabal et al., 2017). However, depending on each specific application with different imposed goals in heart research, weakly coupled problems are also considered and sequentially solved (Frotscher et al., 2016; Duong et al., 2018). On the other hand, decoupled or staggered approaches are suitable for one-way coupling formulation (Usyk et al., 2002; Nash and Panfilov, 2004; Baillargeon et al., 2014; Frotscher et al., 2016). While the effects of the MEF have been widely studied in experiments, the effects in connection with various computational models, especially different passive material models, remain relatively unclear. In this study, the MEF is investigated in combination with various types of passive mechanical laws (nearly incompressible/compressible, polynomial/exponential-type, transversally isotropic/orthotropic material models) in order to study their interaction with the MEF.

Specifically, we want to focus on the interaction of the passive mechanical model with the MEF and how different types of material laws (compressible, incompressible, polynomial, exponential, transversely isotropic, orthotropic) are influencing the overall MEF characteristics. We employ a transversely isotropic and nearly incompressible model, a transversely isotropic compressible model (Göktepe and Kuhl, 2010) and the orthotropic and nearly incompressible Holzapfel-Odgen model (Holzapfel and Ogden, 2009). Our study is based on a left ventricle of a rat heart, whose geometry is reconstructed from high resolution MRI at the University of Erlangen-Nuremberg (Duong et al., 2018). The phenomenological model of Aliev-Panfilov is used to represent the electrical excitation (Aliev and Panfilov, 1996). We investigate the variation in TP evolution and mechanical deformation due to the interaction between the passive mechanical models and the MEF.

## 2. Methods

We briefly introduce the basic electromechanical model and numerical methods used to formulate and solve the boundary value problem of the contracting ventricle. More details on the kinematics and constitutive equations can be found in the Appendix A.

### 2.1. Governing Equations for Cardiac Electromechanics

**Figure 1:**
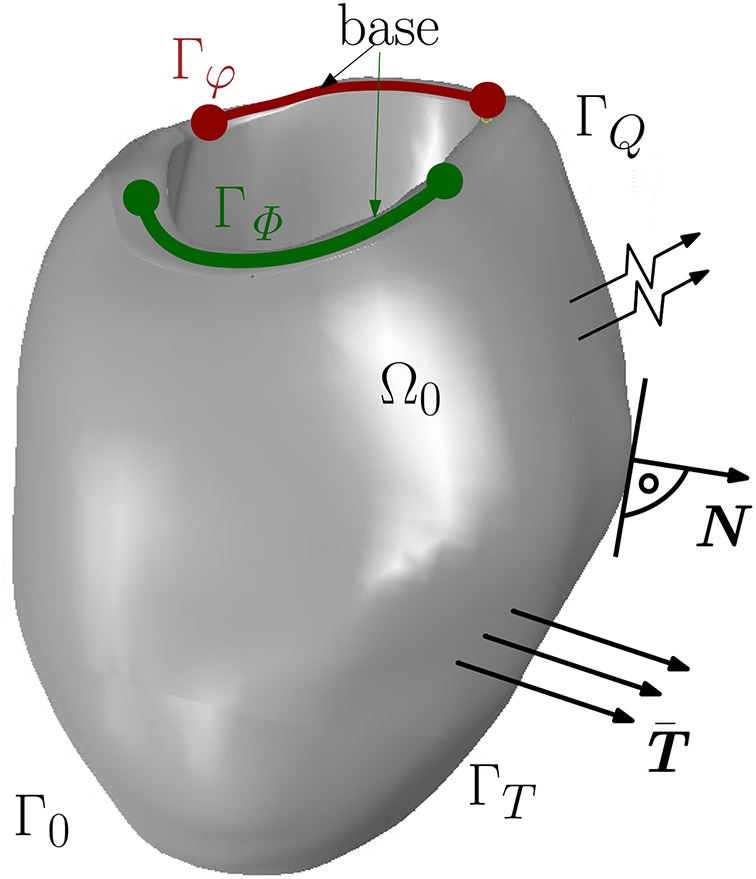
Boundary surface Γ_0_ decomposed into Γ_φ_ and Γ_*T*_ for the mechanical model and Γ_Φ_ and Γ_*Q*_ for the electrical model.

The electromechanics of a left ventricle (LV) (see Figure 1) can be described by two primary field variables, placement ***φ***(***X***, *t*) and AP Φ(***X***, *t*). Thus, two field equations, which govern the state of the material point ***X*** at time *t*, *t* ∈ [*t*_0_, *t*_*f*_] (*t*_0_ and *t*_*f*_ are the initial and final time, respectively), can be formulated. The mechanical field equation is the balance of linear momentum

(1) $$\begin{array}{l} 0=\text{Div}\left[\text{F}\cdot \text{S}\right]+{\text{F}}^{\varphi }\text{ in}\Omega_{0}, \end{array}$$

where **F** is the deformation gradient, **S** is the second Piola-Kirchoff stress tensor and ***F***^Φ^ is the external mechanical body force. The other differential equation describes the spatiotemporal evolution of the AP Φ and can be written as

(2) $$\begin{array}{l} \dot{\Phi }=\text{Div}\left[Q\right]+F^{\Phi }\text{ in}\Omega_{0}. \end{array}$$

The non-linear current term *F*^Φ^ takes into account the electrical excitation, $\dot{\Phi }$ denotes the material time derivative of the AP field, Div[***Q***] represents the diffusion of the AP, whereby ***Q*** describes the electrical flux vector. Together with the boundary conditions, the mechanical and electrical balance equations constitute an initial boundary value problem in the strong form for unknown placement and AP, {***φ***(***X***, *t*), Φ(***X***, *t*)}. The boundary conditions are Dirichlet and Neumann conditions as $\varphi \left(X,t\right)=\bar{\varphi }$ on Γ_φ_, surface traction vector $T\left(X,t\right)=\bar{T}$ on Γ_*T*_ for all *t* ∈ [*t*_0_, *t*_*f*_] and ***φ***(***X***, *t*_0_) = 0 in Ω_0_. For the electrical model, the boundary conditions are $\Phi \left(X,t_{0}\right)=\bar{\Phi }$ on Γ_Φ_ and $Q\left(X,t\right)\cdot N=\bar{Q}$ on Γ_*Q*_ with the unit normal vector ***N*** pointing outwards of surface Γ_*Q*_ (see Figure 1). By the Cauchy stress theorem, we get $\bar{T}=\text{F}\cdot \text{S}\cdot N$.

### 2.2. Mechanical Constitutive Models

By the active stress approach (see e.g., Smith et al., 2004; Göktepe and Kuhl, 2010; Pathmanathan et al., 2010), the stress response of cardiac muscles can be decomposed into a passive and an active part. While the passive part is only governed by the mechanical deformation, the active part takes into account the excitation-induced contraction. Hence, the total second Piola-Kirchhoff stress tensor **S** is written as

(3) $$\begin{array}{l} \text{S}={\text{S}}^{pas}\left(\text{C}\right)+{\text{S}}^{act}\left(f_{0},s_{0},\Phi \right), \end{array}$$

where **C** denotes the right Cauchy-Green deformation tensor, ***f***_0_ the fiber direction and ***s***_0_ the sheet direction in the material configuration. Now, three different models for the passive mechanical response are presented which are utilized to investigate their interaction with the MEF. From the strain energy function Ψ, which is used to describe the mechanics of soft tissues, the associated mechanical constitutive equation reads

(4) $$\begin{array}{l} \begin{array}{c} \text{S}=2\frac{\partial \text{Ψ}}{\partial \text{C}}. \end{array} \end{array}$$

All further derived quantities can be decomposed into their passive (□)^*pas*^ and active part (□)^*act*^.

#### Transversely Isotropic Compressible Model (TIC)

The first passive material model we are utilizing is a transversally isotropic compressible model, which is represented in the form of a polynomial function (see Göktepe and Kuhl, 2010). The passive material stress response is described by a modified neo-Hookean constitutive model in which the basic neo-Hookean model is extended by a transversely isotropic part taking into account the dependency of the material properties in the fiber direction ***f***_0_ at each point ***X*** in the material configuration. The strain energy function can be written as

(5) $$\begin{array}{l} \text{Ψ}={\text{Ψ}}_{iso}+{\text{Ψ}}_{ani} \end{array}$$

(6) $$\begin{array}{l} {\text{Ψ}}_{iso}=\frac{\Lambda }{2}\ln^{2}J+\frac{\mu }{2}\left(I_{1}-3-2\ln\;J\right) \end{array}$$

(7) $$\begin{array}{l} {\text{Ψ}}_{ani}=\frac{1}{2}\vartheta \eta {\left(I_{4f}-1\right)}^{2} \end{array}$$

in terms of the principal strain invariants of the symmetric right Cauchy-Green tensor **C** as *I*_1_ = *tr*(**C**), det(**C**) = *J*^2^, and *I*_4*f*_(**C**) = ***f***_0_ · (**C*****f***_0_), where the Jacobian *J* is defined as *J* = det(**F**). In Equation (5), the two constants Λ and μ in the isotropic part Ψ_*iso*_ are the Lamé parameters, and the passive stiffness of myofibers is denoted by η in the transversely isotropic (so-called anisotropic) part Ψ_*ani*_.

#### Transversely Isotropic and Nearly Incompressible Model (TII)

The second polynomial-type model is basically derived from the above compressible modified neo-Hookean TIC model, but it is rewritten in a form such that it is transversely isotropic and nearly incompressible reading

(8) $$\begin{array}{l} \text{Ψ}={\text{Ψ}}_{iso}+{\text{Ψ}}_{ani}+{\text{Ψ}}_{vol} \end{array}$$

(9) $$\begin{array}{l} {\text{Ψ}}_{iso}=\frac{\mu }{2}\left({\bar{I}}_{1}-3\right) \end{array}$$

(10) $$\begin{array}{l} {\text{Ψ}}_{ani}=\frac{1}{2}\vartheta \eta {\left({\bar{I}}_{4f}-1\right)}^{2} \end{array}$$

in terms of the principal invariants of the isochoric Cauchy-Green tensor $\bar{\text{C}}={\bar{\text{F}}}^{T}\bar{\text{F}}$. The principal isochoric invariants of $\bar{\text{C}}$ are defined as ${\bar{I}}_{1}\left(\bar{\text{C}}\right)=tr\left(\bar{\text{C}}\right)$ and ${\bar{I}}_{4f}\left(\bar{\text{C}}\right)=f_{0}\cdot \left(\bar{\text{C}}f_{0}\right)$. Further, ${\text{Ψ}}_{vol}=\kappa {\left(J-1\right)}^{2}$ is the volumetric energy, where κ tunes the enforcement of incompressibility (we use κ=10^4^ kPa). Similar as in TIC, the anisotropic part of the free energy function Ψ_*ani*_ only occurs if the fibers are under tension $\bar{\lambda }>1$ with $\bar{\lambda }=\sqrt{{\bar{I}}_{4f}}$.

#### Orthotropic and Nearly Incompressible Model (HO)

The third considered model is an exponential-type strain energy function, the Holzapfel-Ogden model (see Holzapfel and Ogden, 2009). In the field of cardiac modeling, the HO model is one of the most suitable choices for describing the passive mechanical response of the myocardial tissues since it can capture the hyperelastic and orthotropic characteristics which have been found in experiments on porcine hearts (see Dokos et al., 2002). In contrast to the polynomial-type models TIC and TII, the HO is based on a strain energy function which is represented by exponentials. The orthotropy at each point ***X*** is characterized by a right-handed set of normalized basis vectors which are determined by the fiber direction ***f***_0_, the sheet direction ***s***_0_ and the orthogonal vector of the sheet plane ***n***_0_ = ***f***_0_ × ***s***_0_. By applying the incompressibility condition to the finite element setting, the HO model is, therefore, split into its isochoric term (in terms of the principal isochoric invariants) and a volumetric part Ψ_*vol*_ (as in TII). The strain energy function reads as

(11) $$\begin{array}{l} \text{Ψ}={\text{Ψ}}_{iso}+{\text{Ψ}}_{ani}+{\text{Ψ}}_{vol}, \end{array}$$

(12) $$\Psi_{iso}=\frac{a}{2b}\exp[b({\bar{I}}_{1}-3)],$$

(13) $$\begin{array}{lll} \Psi_{ani} & = & \sum_{i=f,s}\frac{a_{i}}{2b_{i}}\left\{\exp\left[b_{i}{\left({\bar{I}}_{4i}-1\right)}^{2}\right]-1\right\}\\ \; & \; & +\frac{a_{fs}}{2b_{fs}}\left[\exp(b_{fs}{\bar{I}}_{8fs}^{2})-1\right], \end{array}$$

where *i* ∈ {*f, s*} and the variables *a*, *b*, *a*_*f*_, *b*_*f*_, *a*_*s*_, *b*_*s*_, *a*_*fs*_, *b*_*fs*_ are material constants. While all *a, a*_*f*_, *a*_*s*_, *a*_*fs*_ parameters have the dimension of stress, all *b, b*_*f*_, *b*_*s*_, *b*_*fs*_ are dimensionless.

The active stress response is described in the Appendix A.2.

### 2.3. Electrophysiological Constitutive Models

The nonlinear current source term *F*^Φ^ controlling the AP in Equation (2) is split into two parts as

(14) $$\begin{array}{l} F^{\Phi }=F_{e}^{\Phi }\left(\Phi ,r\right)+F_{m}^{\Phi }\left(\text{C},\Phi \right), \end{array}$$

where $F_{e}^{\Phi }$ expresses the purely electrical part and $F_{m}^{\Phi }$ accounts for the MEF, i.e., a mechanically-induced excitation. The excitation-induced purely electrical part $F_{e}^{\Phi }$ characterizes the effective current, which is generated from the inward and outward flow of ions across the cardiac cell membrane. Meanwhile the stretch-induced mechano-electrical part $F_{m}^{\Phi }$ accounts for the opening of ion channels due to the mechanical deformation. By introducing the non-dimensional and normalized action potential Φ and the non-dimensional time $\bar{t}$, we derive the conversion forms as (Aliev and Panfilov, 1996)

(15) $$\begin{array}{l} \Phi =k_{\phi }\phi -\delta_{\phi },\;t=k_{t}\bar{t}, \end{array}$$

where *k*_ϕ_ and δ_ϕ_ relate the dimensionless action potential ϕ to the physical action potential Φ and *k*_*t*_ the dimensionless time $\bar{t}$ to the physical time *t*. Consequently, the purely electrical term $F_{e}^{\Phi }$ is modeled as

(16) $$\begin{array}{l} \;F_{e}^{\Phi }\left(\Phi ,r\right)=\frac{k_{\phi }}{k_{t}}f_{e}^{\phi }\left(\phi ,r\right),\\ \;f_{e}^{\phi }\left(\phi ,r\right)=c\phi \left(\phi -\alpha \right)\left(1-\phi \right)-r\phi +I, \end{array}$$

where the coefficient α controls the oscillation threshold, *c* is a scaling parameter, *I* is an external stimulus and *r* is the recovery variable which controls the repolarization of the cardiac cell.

#### 2.3.1. Potential Flux

The electrical constitutive equations are also formulated as functions of the deformation gradient and the AP, the material electrical flux reads

(17) $$\begin{array}{l} Q=\text{D}\cdot \nabla \Phi \end{array}$$

with the conductivity tensor $\text{D}=D_{iso}{\text{C}}^{-1}+D_{ani}f_{0}⊗f_{0}/\lambda^{2}$, where *D*_*iso*_ = *Jd*_*iso*_ and *D*_*ani*_ = *Jd*_*ani*_. The parameter *d*_*iso*_ accounts for the isotropic and *d*_*ani*_ for the additional faster conduction along the fiber direction (Costabal et al., 2017).

### 2.4. Mechano-Electrical Feedback

As mentioned before, the electro-mechanical coupling plays an essential role in the cardiac function. A mechanical cell deformation induces electrical current generation. This behavior is observed due to stretch-induced opening of ion channels which induces AP generation. It can be described by the constitutive equation for the electrical source term $F_{m}^{\Phi }$ as

(18) $$\begin{array}{l} \;F_{m}^{\Phi }\left(\text{C},\Phi \right)=\frac{k_{\phi }}{k_{t}}f_{m}^{\phi }\left(\text{C},\phi \right),\\ \;f_{m}^{\phi }\left(\text{C},\phi \right)=\vartheta G_{s}\left(\lambda -1\right)\left(\phi_{s}-\phi \right) \end{array}$$

where *G*_*s*_ denotes the maximum conductance, ϕ is given in Equation (15), ϕ_*s*_ is the dimensionless reversal potential at which there is no net ion flux through the stretch-activated channels, ϑ is a switch function turning the feedback on for λ > 1 and off else. $\lambda =\sqrt{I_{4f}}$ (see Panfilov et al., 2005; Keldermann et al., 2007).

### 2.5. Excitation Stimulated by Deformation in a Plate

In this section, we refer to the benchmark problem performed on a plate using the compressible TIC model to study the effect of the MEF in which excitation wavefronts are observed caused by the deformation-induced excitation of cardiac tissue (Göktepe and Kuhl, 2010; Dal et al., 2013; Cansiz et al., 2015). The orthotropic conductivity is employed in the plate with *d*_*iso*_ = 1.0 mm^2^ ms^−^1 and *d*_*ani*_ = 0.1 mm^2^ ms^−^1, where according to section 2.3.1, the latter accounts for the additional conduction in fiber direction increased by 10% with respect to the other directions (Göktepe and Kuhl, 2009).

**Figure 2:**
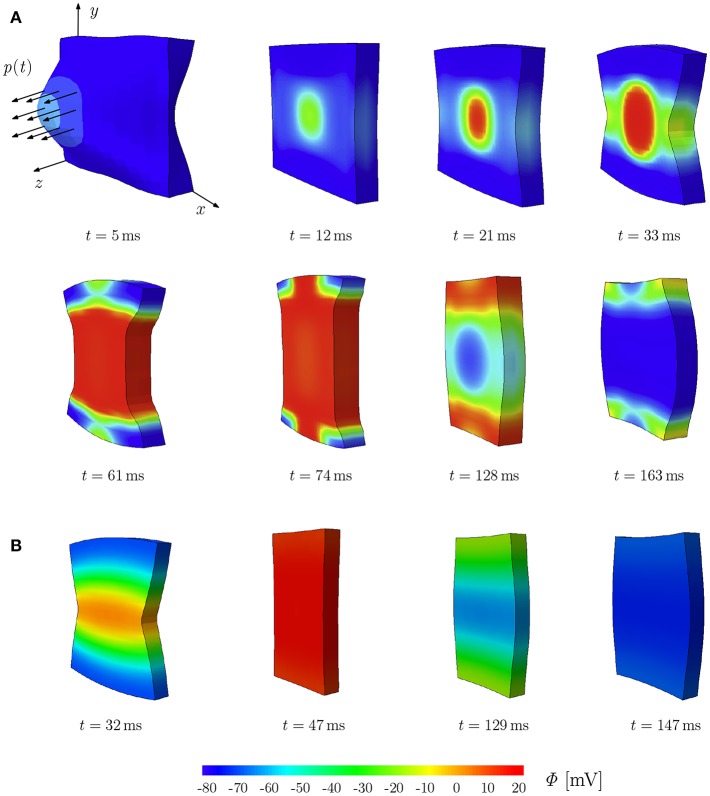
Stretch-induced excitation in a plate; snapshots of the plate colored by the TP. **(A)** Plate of dimension 100 × 100 × 12 mm, *d*_*iso*_ = 1.0 mm^2^ ms^−^1 and *d*_*ani*_ = 0.1 mm^2^/ms^−1^. **(B)** Plate of dimension 10 × 10 × 1.2 mm, *d*_*iso*_ = 0.1 mm^2^ ms^−^1 and *d*_*ani*_ = 0.3 mm^2^/ms^−1^.

To illustrate the deformation-induced excitation in the plate, a mechanical load *p*(*t*) is applied during *t* ∈ [0, 10] ms as shown in Figure 2A. The plate with the dimensions of 100 × 100 × 12 mm is meshed by 21 × 21 × 2 eight-node brick elements. The fiber orientation ***f***_0_ and the sheet plane direction ***s***_0_ are defined in *x*- and *y*-direction, respectively. The compressible model TIC is employed with parameters given in Table A1. The maximum conductance *G*_*s*_ = 15 is utilized to account for the MEF effect. Edge nodes of the plane in the middle at *z* = 6 mm are fixed in *z*-direction. Furthermore, the node at (0, 0, 0) is fixed in *x*- and *y*-directions and the node at (100, 0, 0) in *y*-direction. At the beginning of the simulation, the cellular TP in the plate is set to the resting value Φ = −80 mV. To trigger the MEF, the nodes of a parallelepiped located in the center region of the plate with a dimension of 20 × 20 × 12 mm are subject to an impulsive cyclic loading *p*(*t*) from *t* = 0 to *t* = 10 ms. It linearly reaches its maximum of 0.3 N at *t* = 5 ms and returns to 0 N at *t* = 10 ms.

This loading results in a stretched region in the middle of the plate and hence gives rise to a local depolarization, which then initiates an excitation wave traveling first elliptically (due to orthotropic conductivity, see *t* = 12, 21, and 33 ms) and then unidirectionally along the y-direction. At the same time, the plate shortens in the fiber direction *x* and elongates along the other directions orthogonal to the fibers (*t* = 61 and 74 ms). Since the model is compressible, the whole plate contracts and reduces its volume. The action potential impulse travels to the plate sides and the plate starts to repolarize with a lower potential wave starting in the middle as an elliptical shape (*t* = 128 and 163 ms). While repolarizing, the plate recovers the initial volume due to the relaxation phase of the cardiac muscles. This illustrates the change of TP due to the MEF, which will be discussed in more detail in the next section for different types of mechanical models. It is worth noting that for the TIC model, the applied mechanical load needs to be relatively large in order to cause a visible MEF effect. The same test is performed on a plate of the rat heart dimension (10 × 10 × 1.2 mm) with parameters used in the later simulations (see Table A1). We observed similar behavior; however due to the small dimension of the plate, complete depolarization is reached at *t* = 47 ms (Figure 2B).

### 2.6. Rat Heart Measurements

To generate a 3D left ventricle model of a rat using MRI data, we conducted several experiments on living and healthy rats. More importantly, we also confirm that the ethics committee (Tierschutzgesetz Regierung Unterfranken) approved our experimental protocol and procedures. Further, only the best measurement result was used to reconstruct the 3D rat left ventricle model in the diastolic phase.

## 3. Results

In this section, we investigate the effect of different passive material laws in a strongly coupled electromechanical model of a 3D rat left ventricle.

### 3.1. Parameters of Cardiac Muscles of Rat Heart

**Figure 3:**
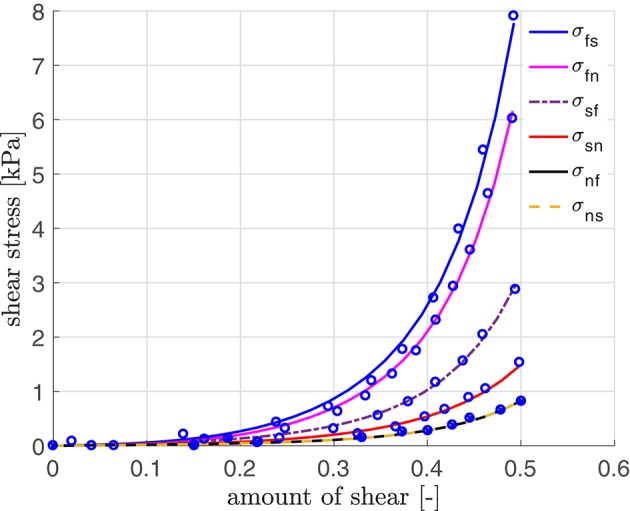
Stress-strain relation with parameters fitted using simple shear data (modified for the rat heart) by Dokos et al. (2002).

The material parameters for our simulation are obtained by curve fitting to experimental data of a porcine heart by Dokos et al. (2002) (see Figure 3; using a scaling factor of 0.5 for rats). The resulting material parameters are displayed in Table A1 (Appendix A.5). The parameters for the electrical and the active model originate partially from our parameter study and partially from work for healthy human hearts (Aliev and Panfilov, 1996; Göktepe and Kuhl, 2010; Baillargeon et al., 2015). We also use *k*_*T*_ = 0.49 kPa mV^−1^ in Equation (24), resulting in $T_{max}^{act}=49$ kPa, which is sufficiently close to the maximum tension value of 45 kPa as obtained in rat experiments (see Niederer et al., 2009). The reversal potential ϕ_*s*_ in Equation (15) is set to 0.6, corresponding to −20 mV, which is in agreement with the physiological value by Kohl et al. (1999).

### 3.2. Rat Left Ventricle

**Figure 4:**
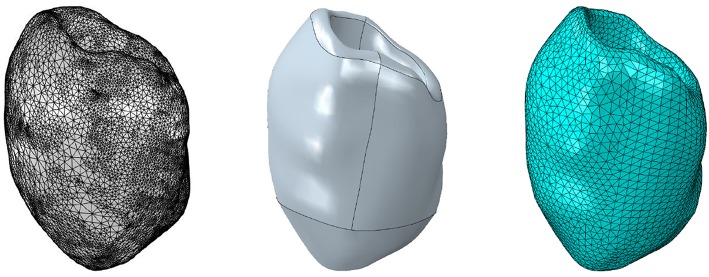
Workflow from MRI images to the finite element mesh of the left ventricle. Left to right: MRI image-segmentation, NURBS model, finite element mesh.

In this section, the interaction of the three different passive material models with the MEF is investigated with regard to the influence of the maximum conductance with *G*_*s*_ = 10 and *G*_*s*_ = 0. For the electromechanical simulation, the parameters given in Table A1 (Appendix A.5) are used. The base of the ventricle is mechanically fixed (see Figure 1). However, different boundary conditions (Baillargeon et al., 2015) can be applied such that the base of the ventricle can slightly translate or twist when it contracts and interacts with surrounding tissues, which greatly support and stabilize the whole heart. The mesh of the 3D solid left ventricle model is generated using 4-node tetrahedral elements based on a high resolution MRI from the Universitätsklinikum Erlangen-Nürnberg. The basic workflow from the MRI images to the finite element mesh can be seen in Figure 4.

**Figure 5:**
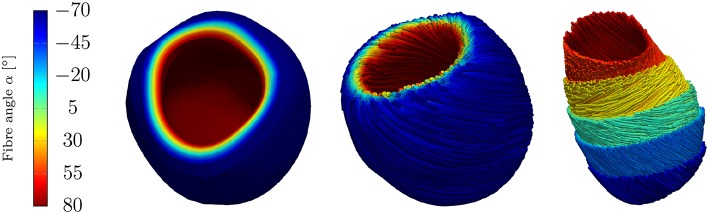
Rat left ventricle with fiber orientations from −70° on the epicardium to +80° on the endocardium with respect to the circumferential direction; **(Left)**: the linear distribution of fiber angles in colors, **(Center)**: fibers (streamlines) on endocardium and epicardium, **(Right)**: fibers on different layers.

The fiber and sheet orientations, which are crucially attributing to the mechanics and electrical conduction system, are assigned for the LV by interpolating the local fiber and sheet directions from the endocardial and epicardial surface such that the model can account for the transmural fiber and sheet directions (Vetter and McCulloch, 1998; Wong and Kuhl, 2014). Although the fiber angles on the endocardium and the epicardium vary largely between different rats (Chen et al., 2003; Hales et al., 2012; Mekkaoui et al., 2012), the fiber angles are chosen as +80° on the endocardium and −70° on the epicardium (see Figure 5).

**Figure 6:**
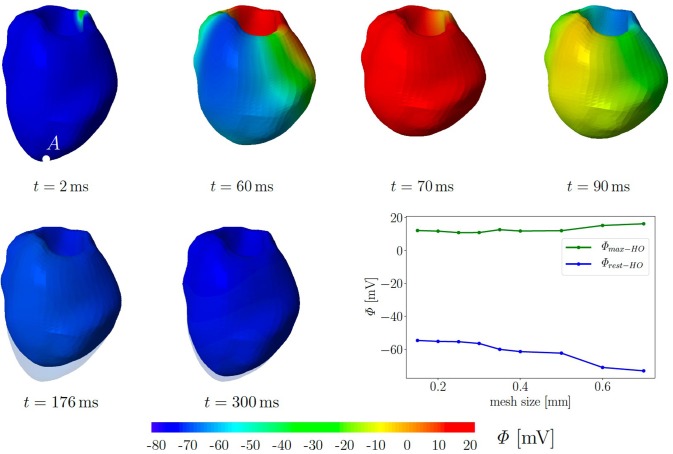
Electromechanics in rat left ventricle with the HO model with MEF and *Gs* = 10. Material parameters are given in Table A1. Excitation by setting seven nodes at the base to Φ = −20 mV for 40 ms. At the end of cardiac cycle (around *t* = 300 ms), the repolarised (resting) LV (blue) is unable to return to its initial shape (light blue region) since the MEF affects the AP and deformation of the LV. Plot at the right bottom shows the relation of maximal and residual potential versus the global mesh size (HO model with MEF and *G*_*s*_ = 50).

For simplicity, we assume a chamber pressure of zero, no electrical flux on the boundary as well as zero surface traction. The computational mesh of the 3D LV consists of 49,769 tetrahedral elements corresponding to the global mesh size of 0.35 mm. Initially, all 10,237 nodes are set to the resting potential Φ_*r*_ = −80 mV. Seven nodes at the base are used to trigger the depolarization in the surrounding cardiomyocytes. They are constrained at Φ = −20 mV for a duration of 40 ms. Subsequently, we solve six fully coupled electromechanical problems for the three different passive material models with and without the MEF (TIC_*G*_*s*_ = 10_, TIC_*G*_*s*_ = 0_, TII_*G*_*s*_ = 10_, TII_*G*_*s*_ = 0_, HO_*G*_*s*_ = 10_, HO_*G*_*s*_ = 0_). We performed simulation on refined meshes up to a mesh size of 0.15 mm. The plot in Figure 6 illustrates that neither the maximal, nor the residual TP show significant changes in regime of smaller meshes. In particular, the resting potential of −80 mV is not reached for all tested mesh sizes.

**Figure 7:**
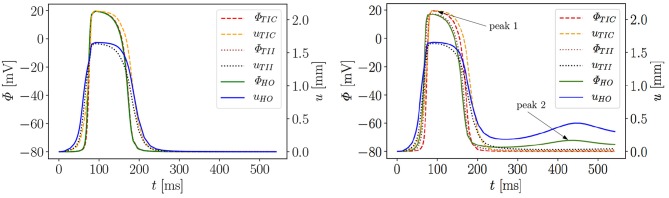
Evolution of AP Φ and displacement *u* of apical node *A* in a rat left ventricle for three models, without MEF **(Left)** and with MEF using *G*_*s*_ = 10 **(Right)**.

Figure 6 exemplarily shows the numerical result for the HO model with the MEF (HO_*G*_*s*_ = 10_)—the location of the stimulus can be seen at the base at *t* = 2 ms. From there, the electrical impulse propagates through the ventricle, see snapshots at *t* = 60 and 70 ms. The entire myocardium contracts at *t* = 90 ms when all ventricular cells are depolarized (in red). After the depolarization, the repolarization follows (*t* = 120, 150, 176, and 300 ms), but the ventricle is unable to return to the initial shape (light blue shadow) even for *t* ≥ 300 ms. This behavior corresponds to a second peak in the AP curve for the HO model (peak 2 above −80 mV) after peak 1 (see Figure 7, right).

Moreover, the AP curve for the TII model with *G*_*s*_ = 10 in the repolarization phase reaches a minimum value of about −78.5 mV for *t* ≥ 300 ms, while the AP curve for the TIC model is able to retrieve the resting potential of −80 mV (see Figure 7, right). The same characteristic behavior can be observed by plotting the apex displacement *u* (Figure 7, right). On the contrary, Figure 7 (left) exhibits no peak 2 in the AP and displacement curves for all three models without MEF (TIC_*G*_*s*_ = 0_, TII_*G*_*s*_ = 0_, HO_*G*_*s*_ = 0_). The LV consequently relaxes to its initial shape. The stretch-induced excitation changes the AP duration, leads to a delayed repolarization and thus the resting potential is reached more slowly. In Figure 7 (left), the AP evolution profiles for the three models are very similar; however, the displacement predicted by the compressible TIC model is larger than the displacements predicted by the TII and HO models. Compared to the AP curves, the displacement of the apex starts before the depolarization. This is caused by the fact that the cardiac cells between the initiated nodes at the base and the considered node at the apex already depolarize and start to contract and hence cause the pre-displacement of the apex.

In summary, it can be stated that both incompressible models yield a different electrical and mechanical response (residual deformation and resting potential not reached at the end of the cardiac cycle) of the LV compared to the compressible TIC model. This raises the question, what exactly causes the difference in the results among the three passive mechanical models. On the one hand, the HO represents an orthotropic material law which is represented by exponentials, while the other two models are transversely isotropic and formulated in terms of polynomials. On the other hand, the HO and TII are both incompressible models, while TII is compressible. To study this more comprehensively, we extended the numerical investigations by performing a parameter study on the maximum conductance *G*_*s*_, which is commonly known as the maximum stretch-activated ion channel conductance or the sensitivity of the electrical current to deformation. Note that diseased hearts can cause abnormal changes in the maximum conductance (Zhang et al., 2014). Thus, this value can be considered as a key variable to study the MEF effects.

**Figure 8:**
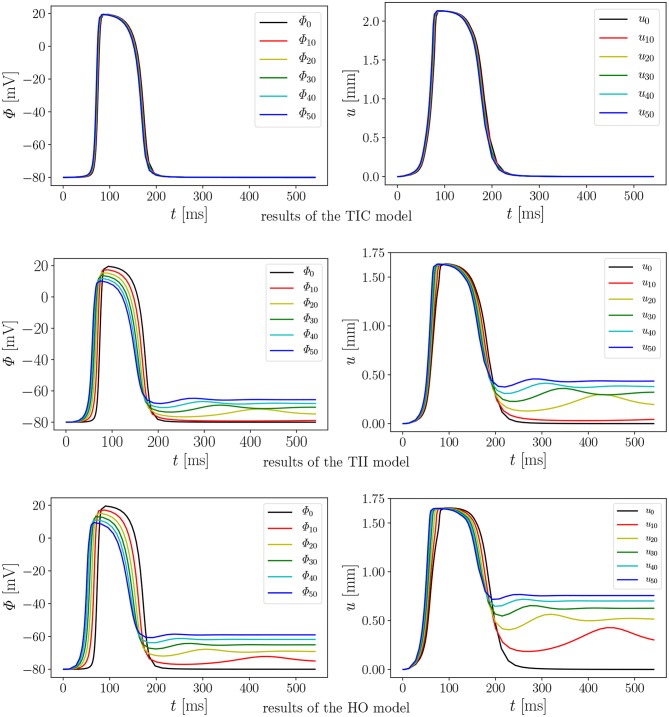
Evolution of AP Φ **(Left)** and displacement *u* **(Right)** of node *A* with varying *G*_*s*_ in a rat left ventricle for the three material models.

We performed 18 simulations, which differ in the passive material model (TIC, TII, HO) and the maximum conductance (*G*_*s*_ = 0, 10, 20, 30, 40, and 50). Instead of performing a benchmark problem study using a simple cube, bar or beam, the effect is directly studied in a 3D LV model. The result of the AP evolution and the displacement are shown in Figure 8. The TIC model shows an almost independent behavior with respect to the strength of the MEF [see AP and displacement evolution Figure 8 (top, left, and right)]. The polynomial, transversely isotropic and incompressible material law TII (middle, left, and right) and the exponential, orthotropic and incompressible material law HO (bottom, left, and right) exhibit a high sensitivity to the strength of the MEF. The observed characteristics in Figure 7 (early depolarization, delay in the late repolarization phase, decrease of the maximum AP) are intensified with a higher maximum conductance.

## 4. Discussion

As observed in the last section, the influence of the MEF on the overall electrophysiological and mechanical behavior significantly depends on the passive material model. In the following section, we want to discuss the differences and possible sources or causes. The main differences of the three different passive material models can be summarized as follows: (i) compressible vs. incompressible, (ii) polynomial vs. exponential, (iii) orthotropic vs. transversely isotropic. Their respective results will be evaluated and compared here. Referring to the following explication, we introduce the notation: □_*max*−*a*_ and □_*rest*−*a*_ for values of the material *a* at peak 1 (e.g., the maximal value of the AP or displacement) and at peak 2 close to the resting potential −80 mV and zero deformation (see Figure 7, right), respectively. We define the difference of a value at *G*_*s*_ = *x* to that at *G*_*s*_ = 0 as $\Delta {□}_{i-a}^{x}=|{□}_{i-a}\left(G_{s}=0\right)-{□}_{i-a}\left(G_{s}=x\right)|$, where *i* ∈ {*max, rest*}.

**Figure 9:**
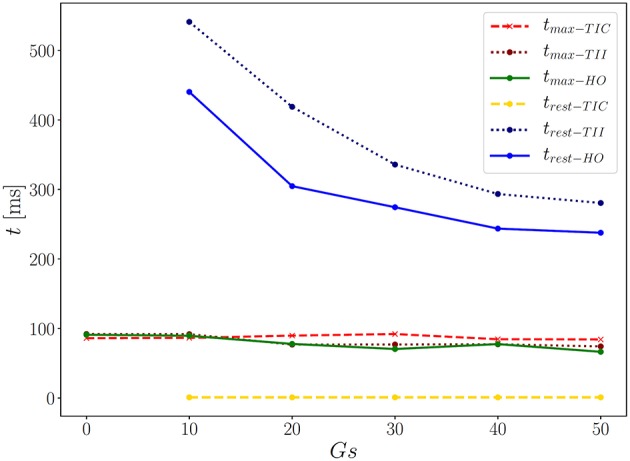
Time points for corresponding two AP peaks for the three models.

First we note that the MEF is capable of speeding up the electrical impulse propagating in the LV (Figure 9). For the compressible TIC model, it is clearly observed that the MEF has negligible effects on the electrophysiology and mechanics. The largest change in time for the AP peak is $\Delta t_{max-TIC}^{50}$ = 5 ms while there is no peak 2 and thus $t_{rest-TIC}^{x}=0$ for all values *x* ∈ {0, 10, 20, 30, 40, 50} (see also peak values in Figure 9). In contrast to that, we observe a significant effect of the MEF (or maximum conductance) for the incompressible models TII and HO. Figure 8 indicates that for higher value of *G*_*s*_, larger changes in AP and displacement curves can be observed. The largest changes in peak time for AP are Δ $t_{max-TII}^{50}$ = 18 ms and Δ $t_{max-HO}^{50}$ = 23 ms for the TII and HO models, respectively. The conduction velocity is apparently increased with *G*_*s*_ which is in good agreement with the findings in a study by Costabal et al. (2017). Similar observations have also been made in the numerical results by Amar et al. (2018), in which the AP was influenced by the MEF for different stretch levels for examples of a ventricle and a single cardiomyocyte.

**Figure 10:**
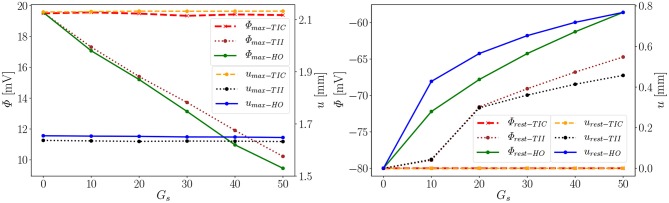
Φ_*max*_ and *u*_*max*_ of peak 1 **(Left)**, Φ_*rest*_ and *u*_*rest*_ of peak 2 **(Right)** for the three models (Φ_*rest*−*TIC*_ curves invisible since *u*_*rest*−*TIC*_ lies on top of it).

Second, depending on the material model, the MEF influences AP Φ and displacement *u* in the late repolarization phase. For the compressible model TIC, no change in peak 2 for the AP and displacement curves (Δ $\Phi_{rest-TIC}^{x}$ = 0 mV and Δ $u_{rest-TIC}^{x}$ = 0 mm) for all values *x* ∈ {0, 10, 20, 30, 40, 50} can be observed. At the same time, there are significant changes in peak 2, which can be observed in the curves for the TII and HO model. For example, the largest changes are introduced by *G*_*s*_ = 50 such as $\Delta \Phi_{rest-TII}^{50}=16$ mV and $\Delta \Phi_{rest-HO}^{50}=22$ mV, whereas $\Delta u_{rest-TII}^{50}=0.45$ mm and $\Delta u_{rest-HO}^{50}=0.79$ mm (see Figure 10, right). The maximum value of the AP Φ and displacement *u* for peak 1 for (*G*_*s*_ = *x*) for all values *x* ∈ {0, 10, 20, 30, 40, 50} are depicted in Figure 10 (left). While varying the maximum conductance *G*_*s*_, the compressible model leads to an almost unchanged electrophysiology and mechanics, the nearly incompressible models show a stronger effect of the MEF on the electrophysiology (early depolarization, reduced maximum AP, increased conduction velocity, delayed repolarization) and consequently result in a significantly higher relaxation displacement of the LV.

### Compressible vs. Incompressible

**Figure 11:**
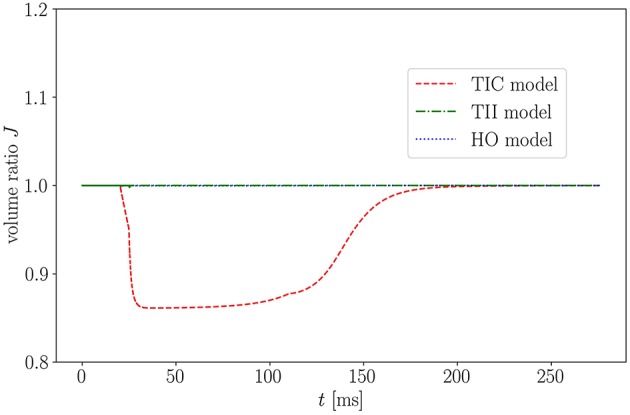
Volume ratio *J* of isotonic contraction of a cube over time for the three models.

One important characteristic which influences the impact of the MEF on the overall model behavior is the level of compressibility. The compressible TIC model undergoes a significant volume change (compressible medium) during the active contraction phase, which in turn leads to a reduced overall strain/stretch. This is visualized in Figure 11 showing the isotonic contraction of a cube for the three different material models.

**Figure 12:**
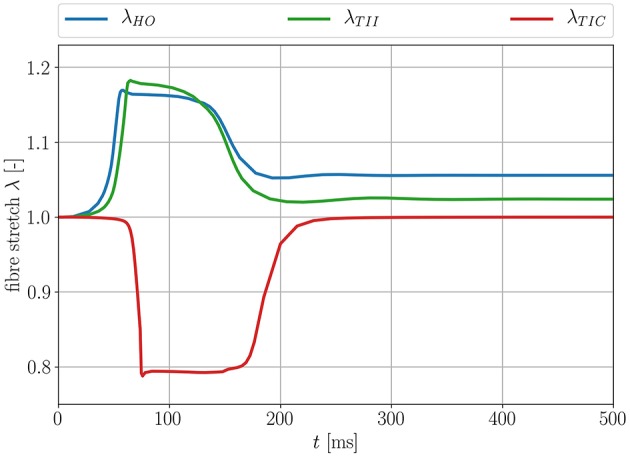
Fiber stretch λ for an apical node for the three different passive material models.

As expected, when considering the volume ratio curves for the three models in isotonic contraction, the volume ratio during the time course of the cardiac cycle for the compressible TIC model decreases ineluctably, while the volume for the HO and TII models remains constant. By plotting the fiber stretch λ of the same apical node of the LV for the HO, TII and TIC model, we observe the fiber stretch λ < 1 (compression) for the TIC model and the fiber stretch λ > 1 for the HO and TII models (see Figure 12).

**Figure 13:**
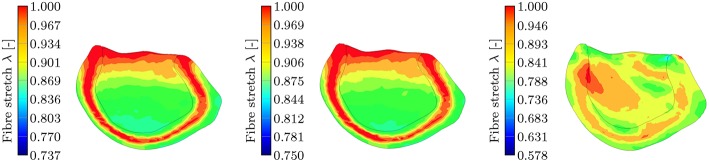
Section view of the LV during the contraction phase at *t* = 90 ms for the three different passive material models. The snapshots show the transmural fiber stretch λ. The color code is limited to the maximum fiber stretch of 1 and thus all dark red regions show fiber stretch λ ≥ 1. The maximum global fiber stretch for the three different models reads as: $\lambda_{HO}^{max}=1.188$, $\lambda_{TII}^{max}=1.204$, $\lambda_{TIC}^{max}=1.024$. **(Left)**: transmural fiber stretch—HO, **(Middle)**: transmural fiber stretch—TII, **(Right)**: transmural fiber stretch—TIC.

This leads to the fact that in this particular region, the MEF is active for the TII and HO models and inactive for the TIC model (see Equation 18, λ has to be >1 for the switch function ϑ to be non-zero). Furthermore, the fiber angles transmurally vary and thus the local change in fiber direction leads to an increased fiber stretch through the wall (the expansion of the material in sheet and normal direction close to the boundaries leads to a fiber stretch in the middle layers; see Figure 13, left, center).

This behavior is characteristic for the incompressible TII and HO models. The TII model compensates this phenomenon due to the compressibility (volume change), and thus the expansion of the material in the sheet and normal direction is smaller, which finally induces no or insignificant stretches to the fibers in the middle layer (see Figure 13, right). For the TII and HO model, the high fiber stretch in the middle layers during the repolarization phase effectively causes a strong MEF current. In a real healthy rat left ventricle, the MEF effect might be caused by a strong mechanical stimulus when the AP reaches the threshold value. However, it is observed mainly in diseased cases (Kamkin et al., 2000). The MEF can alter the AP or shorten refractory periods in canine left ventricle and atrium, as reported in Franz (1996). Stretch-activated polarization and fibrillation can happen at a low stretch in a rat atrium after the infarction of the left ventricle (Kamkin et al., 2000). Moreover, it has been observed in our simulation of the LV as well as for cubes, plates and other 2D examples in Costabal et al. (2017), that the MEF is capable of altering the AP and increasing the conduction velocity. However, the phenomenon of the residual deformation (non-relaxed equilibrium between the AP generation and the fiber stretches in the late repolarization phase) is rather unrealistic in the healthy left ventricle simulation.

### Polynomial vs. Exponential

In addition to the discussed differences due to the level of compressibility, the exponential and polynomial form of the passive material law plays a significant role concerning the overall MEF behavior. To clarify this, we performed the numerical tests for the HO and TII models with the same bulk modulus of κ = 10^4^ kPa, which means that these models have the same level of compressibility. The compressibility value is reasonable in our simulations as it is only slightly larger compared to the *in vivo* measurements by Hassaballah et al. (2013). To compare the influence of the polynomial material law TII and the exponential material law HO, we evaluate the differences in Figures 8, 10, 12, 13.

In the depolarization phase, the HO and TII models show an almost similar behavior concerning the AP and displacement curves even if the observed phenomenon is slightly more prominent for the HO model (see Figures 10, 8, left). Nevertheless, in the late repolarization phase, the AP and displacement curves significantly differ between the HO and TII models (see Figure 10, right). It is worth noting that both models undergo a comparable maximum stretch level (see Figure 13, left, center). Thus the question arises regarding where the significant differences in the late repolarization come from.

Since the same bulk modulus is used, both models generate the same amount of active stress and result in relatively comparable displacements (see Figure 13, left, center). Therefore, the difference can only be explained by the fact that the HO model induces larger strain/stretch in the late repolarization phase and in turn produces a higher MEF current compared to the TII model. Figure 12 exposes that the HO model has a steeper increase in the fiber stretch in the depolarization phase (which explains the faster depolarization compared to the TII model) and a slower change of the fiber stretch in the repolarization phase (which explains the higher stretch in the late repolarization phase). In other words, starting from the same deformation level during the contraction for the HO and TII models, the cardiac tissue relaxes in the repolarization phase, in which the exponential-type stress-strain relation of the HO model responds to the decreasing active fiber tension with a smaller change in fiber stretch compared to the TII model. Finally, this leads to a high MEF current in certain regions, which almost reached their resting potential.

### Transversely Isotropic vs. Orthotropic

As we discussed in the previous paragraph, the main difference in the AP and displacement for the HO and TII models originate in the representation of the material law. Nevertheless, the incompressibility condition with the associated stretches along the sheet and sheet normal direction at the boundaries [see Figure 13 (left, center), fiber stretch λ < 1, and thus the compression in fiber direction has to be compensated by stretch in the sheet and sheet normal direction] leads to a stretch in fiber direction in the middle layers. As the fiber stretch λ depends on the material properties in sheet and sheet normal direction, there also exists a difference between a transversely isotropic and an orthotropic material law concerning the MEF behavior. To further investigate the difference, one would have to vary the passive material properties in sheet or sheet normal direction for the same material model (note that for the special case of transversal isotropy, the properties in sheet and sheet normal direction are the same) in order to eliminate the influence of the type of material model (polynomial and exponential). However, this goes beyond the scope of this study. In our case, the difference between the orthotropic material model HO and the transversely isotropic material model TII seems to be rather small as the overall fiber stretch levels are similar.

### 4.1. Limitations of the Work

In the following, we want to briefly discuss the limitations and assumptions for this study. Firstly, we ignore the possible influence of the right ventricle and other components which also contribute to the overall model behavior. Secondly, the electromechanical model uses several parameters, which are directly adapted or come from other studies on human and porcine hearts. Furthermore, the electrophysiological model is an obvious simplification of the actual electrophysiology of the heart. There exist different kinds of cardiac cells in the heart with different contractility and conductivity. In particular, the fast conducting Purkinje fibers need to be included in order to improve the choreographed depolarization and repolarization of the heart. However, the simple electrophysiological model is to be preferred for our application whereby a complex rat heart support system will be developed in order to compensate the function of the pathological heart. Further, more detailed experimental data, especially for rats, about the electrical and active model including the determination of the active muscle tension, transmembrane potential and maximum conductance, *G*_*s*_ are required. Measurements to identify the passive material parameters for rats would be desired to further improve the accuracy of the model. Moreover, the fixed base in the simulation model simplifies the actual support of the heart in the body (e.g., connective tissue, atria) and an advanced boundary condition should be applied for future studies. Further investigations are necessary to evaluate the influence of the specific morphology of the heart (different fiber distributions). Additionally, to more precisely describe the exact influence and differences of transversely isotropic and orthotropic material laws concerning the MEF behavior, a parameter study about the level of orthotropy is necessary.

## 5. Conclusion

In this paper, we focus on the interaction of passive mechanical models with the MEF and how different types of material laws (compressible, incompressible, polynomial, exponential, transversely isotropic, and orthotropic) influence the overall MEF characteristics. We employ a transversely isotropic and nearly incompressible model (TII), a transversely isotropic compressible model (TIC) (Göktepe and Kuhl, 2010) and the orthotropic and nearly incompressible Holzapfel-Odgen model (HO) (Holzapfel and Ogden, 2009). We investigate the variation in AP evolution and mechanical deformation due to the interaction between the passive mechanical models and the MEF.

The interaction between the passive models and the MEF is discussed through a computational study of a rat LV, whereby the following findings are obtained: (i) compressibility: the transversely isotropic material law (TIC) predicts a significantly smaller fiber stretch (compression almost everywhere in the LV) and thus leads to a nearly unrecognizable change in the overall MEF behavior (change in electrophysiology and mechanical contraction); additionally, for the incompressible models, we observe a residual deformation caused by a non-relaxed equilibrium; (ii) polynomial vs. exponential material laws: due to the exponential strain energy function, the HO model shows a faster temporal change of the fiber stretch in the depolarization phase (leading to a faster depolarization compared to the TII model; higher MEF current) and at the same time slower temporal change of the fiber stretch in the late repolarization phase; (iii) transversely isotropic vs. orthotropic: the incompressibility condition with the associated stretches along the sheet and sheet normal direction on the boundaries [see Figure 13 (left, center), fiber stretch λ < 1 and thus the compression in fiber direction has to be compensated by stretch in the sheet and sheet normal direction] leads to a stretch in fiber direction in the middle layers. As the fiber stretch λ depends on the material properties in the sheet and sheet normal direction, there also exists a difference in the electromechanical behavior and the MEF between a transversely isotropic and an orthotropic material law concerning the MEF behavior.

Obviously, the type of passive material model plays a key role in defining the MEF behavior in a fully coupled electromechanical model. It has to be further investigated which of the considered models reflect the cardiac tissue best concerning the overall MEF behavior.

## Ethics Statement

The experimental protocol and procedures were approved by the ethics committee (Tierschutzgesetz Regierung Unterfranken).

## Author Contributions

MD mainly contributed to this paper concerning the manuscript, modeling, and simulation. DH contributed through the geometric and fiber modeling, simulation as well as the revision of the paper. MA, SD, and SL contributed through the conception and design of the study. MA provided the MRI data and the segmentation.

### Conflict of Interest Statement

The authors declare that the research was conducted in the absence of any commercial or financial relationships that could be construed as a potential conflict of interest.

## Figures and Tables

**Figure A1:**
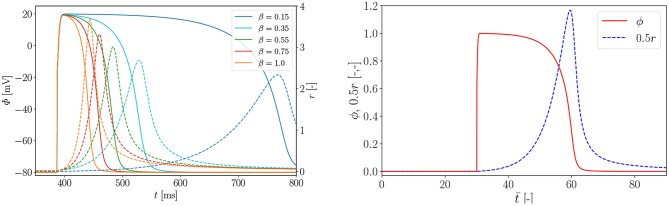
Left: parameter study for rat electrophysiology AP (solid) and recovery variable (dashed); Right: evolution of normalized AP ϕ and recovery variable 0.5*r* over normalized time $\bar{t}$. Parameters α = 0.01, γ = 0.002, β = 0.55, *c* = 8, μ_1_ = 0.2 and μ_2_ = 0.3 and an external excitation *I* = 30 at $\bar{t}=30$ to trigger cardiac cells.

**Figure A2:**
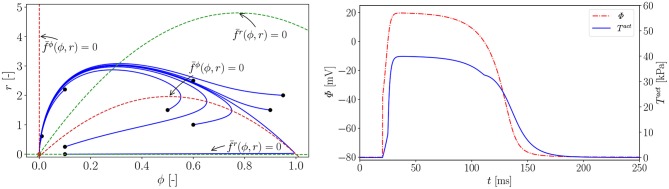
Left: phase plane; the nullclines ${\bar{f}}^{r}$ and ${\bar{f}}^{\phi }$ (dashed lines) are functions of ϕ and $\bar{t}$ and guide all the solution trajectories (solid lines) to the equilibrium point from different starting points (•); Right: Active fiber tension *T*^*act*^ (Φ). Parameters: $\epsilon_{0}=0.1\;{\text{ms}}^{-1}$, $\epsilon_{\infty }=1.0\;{\text{ms}}^{-1}$, $\tilde{\Phi }=0.0\;\text{mV}$, ξ = 0.5 mV^−1^, *k*_*T*_ = 0.40 kPa and Φ_*r*_ = −80 mV. Electrical characteristics as in Figure A1.

## A. Appendix

### A.1. Kinematics and Finite Element Approximation

The deformation gradient is defined as:

(A1) $$\begin{array}{l} \text{F}=\frac{\partial \varphi \left(X,t\right)}{\partial X}=\nabla \varphi \left(X,t\right)\text{ with }J=\text{det }\text{F}>0, \end{array}$$

where the determinant of the deformation gradient *J* is also known as the volume ratio and has to be positive as the body is impenetrable. Further, the right Cauchy-Green tensor **C** can be introduced as **C** = **F**^*T*^**F**. To enforce the material incompressibility conditions (*J* = 1) in a framework of a finite element setting, the deformation gradient can be split into two parts $\text{F}=\left(J^{1/3}\text{I}\right)\bar{\text{F}}$, where **I** is the identity tensor. Specifically, *J*^1/3^**I** describes purely volumetric deformation whereas $\bar{\text{F}}$ denotes the purely isochoric deformation ($\bar{J}=det\left(\bar{\text{F}}\right)=1$).

Employing the Galerkin procedure, the residuals (2) and (1) are multiplied with the scalar- and vector-valued test functions δΦ and δ***φ*** which satisfy δΦ = 0 on Γ_Φ_ and δ***φ*** = *0* on Γ_***φ***_, respectively. These resulting expressions are further reformulated by using integration by parts and the Gauss theorem over the body Ω_0_ such that the weak forms are obtained as:

(A2) $$\begin{array}{l} \begin{array}{c} \int_{\Omega_{0}}\delta \Phi \dot{\Phi }\text{d}V+\int_{\Omega_{0}}\nabla \left(\delta \Phi \right)\cdot Q\text{d}V\\ \;-\int_{\Omega_{0}}\delta \Phi F^{\Phi }\text{d}V-\int_{\Gamma_{Q}}\delta \Phi \bar{Q}\text{d}a\;\dot{=}\;0,\\ \int_{\Omega_{0}}\nabla \left(\delta \varphi \right):\left[\text{F}\cdot \text{S}\right]\text{d}V-\int_{\Omega_{0}}\delta \varphi \cdot F^{\varphi }\text{d}V-\int_{\Gamma_{T}}\delta \varphi \cdot \bar{T}\text{d}a\;\dot{=}\;0. \end{array} \end{array}$$

Further, the Dirichlet boundary conditions prescribe the state of the respective surface points to be $\bar{\varphi }$ and $\bar{\Phi }$. The Neumann boundary conditions prescribe the surface traction $\bar{T}$ and the surface flux term $\bar{Q}=Q\cdot N$. ***F***^Φ^. All quantities of the boundary conditions are supposed to be given. In addition, in our problems $\bar{T}$, $\bar{Q}$ and ***F***^Φ^ are independent of deformation and AP. In what follows, the notations are introduced: the material time derivative as $\left\{\dot{□}\right\}$ = d{□}/d*t* and the material divergence and gradient as Div{□} = ∂{□}/∂***X*** : **I** and ∇{□} = ∂{□}/∂***X***. The equation of motion expresses the quasi-static force equilibrium and has to hold for all points ***X*** in Ω_0_. In the following, we will review how to derive the main terms in the (A2).

### A.2. Mechanical Constitutive Models

#### A.2.1. Transversely Isotropic Compressible Model (TIC)

The passive second Piola-Kirchhoff stress **S**^*pas*^ can be derived from the strain energy (5) as:

(A3) $$\begin{array}{l} {\text{S}}^{pas}\left(\text{C}\right)=2\frac{\partial \text{Ψ}}{\partial \text{C}}={\text{S}}_{iso}\left(\text{C}\right)+{\text{S}}_{ani}\left(\text{C}\right),\\ \;{\text{S}}_{iso}\left(\text{C}\right)=\left(\Lambda \ln\;J-\mu \right){\text{C}}^{-1}+\mu \text{I},\\ {\text{S}}_{ani}\left(\text{C}\right)=+2\vartheta \eta \left(I_{4f}-1\right)f_{0}⊗f_{0}. \end{array}$$

#### A.2.2. Transversely Isotropic and Nearly Incompressible Model (TII)

The passive second Piola-Kirchhoff stress **S**^*pas*^ can be derived from (8) as follows:

(A4) $$\begin{array}{l} {\text{S}}^{pas}\left(\text{C}\right)=2\frac{\partial \text{Ψ}}{\partial \text{C}}={\text{S}}_{iso}\left(\text{C}\right)+{\text{S}}_{ani}\left(\text{C}\right),\\ \;{\text{S}}_{iso}\left(\text{C}\right)=\mu \left(J^{-2/3}\text{I}-\frac{1}{3}{\bar{I}}_{1}{\text{C}}^{-1}\right),\\ {\text{S}}_{ani}\left(\text{C}\right)=2\vartheta \eta \left({\bar{I}}_{4f}-1\right)\left(f_{0}⊗f_{0}-\frac{1}{3}{\bar{I}}_{4f}{\text{C}}^{-1}\right). \end{array}$$

#### A.2.3. Orthotropic and Nearly Incompressible Model (HO)

Based on (11), the passive second Piola-Kirchhoff stress is given as ${\text{S}}^{pas}={\text{S}}_{iso}+{\text{S}}_{ani}$, where

(A5) $$\begin{array}{c} \;{\text{S}}_{iso}\left(\text{C}\right)=a\exp\left[b\left({\bar{I}}_{1}-3\right)\right]\left(J^{-2/3}\text{I}-\frac{1}{3}{\bar{I}}_{1}{\text{C}}^{-1}\right),\\ {\text{S}}_{ani}\left(\text{C}\right)=\underset{i=f,s}{\sum }2\left({\bar{I}}_{4i}-1\right)a_{i}\exp\left[b_{i}{\left({\bar{I}}_{4i}-1\right)}^{2}\right]\left(i_{0}⊗i_{0}-\frac{1}{3}{\bar{I}}_{4i}{\text{C}}^{-1}\right)\\ \;+{\bar{I}}_{8fs}a_{fs}\exp\left(b_{fs}{\bar{I}}_{8fs}^{2}\right)\left[\left(f_{0}⊗s_{0}+s_{0}⊗f_{0}\right)-\frac{2}{3}{\bar{I}}_{8fs}{\text{C}}^{-1}\right]. \end{array}$$

#### A.2.4. Transversely Isotropic Active Stress Response

Taking into account ***f***_0_ only, the active second Piola-Kirchhoff stress is defined as follows:

(A6) $$\begin{array}{l} {\text{S}}^{act}\left(f_{0},\Phi \right)=T^{act}\left(\Phi \right)f_{0}⊗f_{0}. \end{array}$$

The transversely isotropic characteristic is realized such that the magnitude of the active fiber tension *T*^*act*^(Φ), which is driven by the AP, only has an effect along the material fiber orientation ***f***_0_.

#### A.2.5. Orthotropic Active Stress Response

When both ***f***_0_ and ***s***_0_ are considered for active contraction, the stress is written as:

(A7) $$\begin{array}{l} {\text{S}}^{act}\left(f_{0},s_{0},\Phi \right)=T^{act}\left(\Phi \right)\left[\nu_{ff}f_{0}⊗f_{0}+\nu_{ss}s_{0}⊗s_{0}\right], \end{array}$$

where ν_*ff*_ and ν_*ss*_ are weighting factors. Furthermore, *T*^*act*^(Φ) is modeled via the evolution equation as *Ṫ*^*act*^ = *T*(Φ, *T*^*act*^) in Nash and Panfilov (2004). According to the local ordinary differential equation of the active muscle traction, *T*^*act*^ can be treated as an internal variable and locally updated on the integration point level. The evolution equation for the muscle traction is herein used for both active tension models and reads:

(A8) $$\begin{array}{l} {\dot{T}}^{act}=\epsilon \left(\Phi \right)\left[k_{T}\left(\Phi -\Phi_{r}\right)-T^{act}\right] \end{array}$$

with its sensitivity with respect to the action potential.

(A9) $$\begin{array}{l} \frac{\partial {\text{S}}^{act}}{\partial \Phi }=\partial_{\Phi }T^{act}\left(\Phi \right)\left[\nu_{ff}f_{0}⊗f_{0}+\nu_{ss}s_{0}⊗s_{0}\right], \end{array}$$

where *k*_*T*_ specifies the saturated value of *T*^*act*^(Φ), and Φ_*r*_ is the resting potential, where no new tension is evoked. Usually for cardiac cells Φ_*r*_ = −80mV, and ϵ(Φ) represents the switch function which creates the typical cardiac cell performance via:

(A10) $$\begin{array}{l} \epsilon \left(\Phi \right)=\epsilon_{0}+\left(\epsilon_{\infty }-\epsilon_{0}\right)\exp\left[-\exp\left(-\xi \left(\Phi -\tilde{\Phi }\right)\right)\right]. \end{array}$$

The special behavior can be adjusted by the parameters ϵ_0_ and ϵ_∞_, which regulate the limitation values $\tilde{\Phi }$ denoting the phase shift and ξ controlling the transition rate from ϵ_0_ to ϵ_∞_. The impact of the active fiber tension on the active stress along the fiber direction and in sheet direction are controlled by ν_*ff*_ and ν_*ss*_, respectively. Equation (A8) is approximately solved for the internal variable *T*^*act*^. The temporal discretization of the time derivative reads ${\dot{T}}^{act}\approx \frac{T^{act}-T_{n}^{act}}{\Delta t}$. Here, *T*^*act*^ approximates the active fiber tension at time *t*_*n*+1_, while $T_{n}^{act}$ approximates the active fiber tension at *t*_*n*_. Therefore, the residual is formed as follows:

(A11) $$\begin{array}{l} R^{T}=T^{act}-T_{n}^{act}-\Delta t\epsilon \left(\Phi \right)\left[k_{T}\left(\Phi -\Phi_{r}\right)-T^{act}\right]\dot{=}0. \end{array}$$

This equation can directly be solved for *T*^*act*^ by restructuring into

(A12) $$\begin{array}{l} T^{act}\left(\Phi \right)=\frac{1}{1+\Delta t\epsilon \left(\Phi \right)}\left[T_{n}^{act}+\Delta t\epsilon \left(\Phi \right)\left[k_{T}\left(\Phi -\Phi_{r}\right)\right]\right]. \end{array}$$

The sensitivity $\partial_{\Phi }T^{act}\left(\Phi \right)$ of the active stress to the transmembrane potential Φ in (A9) results from its derivation as:

(A13) $$\begin{array}{l} \partial_{\Phi }T^{act}\left(\Phi \right)=\frac{\Delta t}{1+\Delta t\epsilon \left(\Phi \right)}[\epsilon^{\prime }\left(\Phi \right)[\left(k_{T}\left(\Phi -\Phi_{r}\right)-T^{act}\right]\\ +\epsilon \left(\Phi \right)k_{T}] \end{array}$$

and the derivative of the switch function is calculated as:

(A14) $$\begin{array}{l} \epsilon^{\prime }\left(\Phi \right)=\frac{\partial \epsilon \left(\Phi \right)}{\partial \Phi }=\xi \left[\epsilon \left(\Phi \right)-\epsilon_{0}\right]\exp\left[-\xi \left(\Phi -\tilde{\Phi }\right)\right]. \end{array}$$

The sensitivities of potential flux d_**C**_***Q*** and the stress tensor with respect to the deformation d_**C**_**S** can be evaluated as

(A15) $$\begin{array}{l} {\text{d}}_{\text{C}}Q=\{D_{iso}\frac{1}{2}\left[{\text{C}}^{-1}\bar{⊗}{\text{C}}^{-1}\right]+D_{ani}I_{4f}^{-2}\left(f_{0}⊗f_{0}\right)⊗(f_{0}\\ ⊗f_{0})\}\cdot \nabla \Phi . \end{array}$$

### A.3. Electrophysiological Model

In (16), *r* is the recovery variable, whose evolution is governed by the local ordinary differential equation known as the Aliev-Panfilov (Aliev and Panfilov, 1996) model, which can capture all major characteristics of the cardiac electrophysiology.

(A16) $$\begin{array}{l} \dot{r}=f^{r}=\left[\gamma +\frac{\mu_{1}r}{\mu_{2}+\phi }\right]\left[-r-c\phi \left(\phi -\beta -1\right)\right], \end{array}$$

where *f*^*r*^ is the source term for *r* and the variables μ_1_, μ_2_, β and γ are additional material parameters. While the coefficient term $\left[\gamma +\frac{\mu_{1}r}{\mu_{2}+\phi }\right]$ is a weighting factor, β controls the AP duration or effective refractory period (Costabal et al., 2016).

Considering the boundary value problem (A2), *r* can be also treated as an internal variable. In order to solve the internal evolution equation (29), the implicit Euler method is used. This requires a temporal discretization of the time derivative $ṙ\approx \frac{r-r_{n}}{\Delta t}$. Hence, the residual can be introduced as:

(A17) $$\begin{array}{l} R^{r}=r-r_{n}-\Delta tf^{r}\left(\phi ,r\right)\;\dot{=}\;0. \end{array}$$

Using its linearization the local update equation for variable *r* can be achieved as:

(A18) $$\begin{array}{l} r\leftarrow r-{\left(\partial_{r}R^{r}\right)}^{-1}R^{r}\\ \text{with }\partial_{r}R^{r}=1+\Delta t\left[\gamma +\frac{\mu_{1}}{\mu_{2}+\phi }\left[2r+c\phi \left(\phi -\beta -1\right)\right]\right]. \end{array}$$

This is solved iteratively using the Newton-Raphson method.

In Figure A1 (left), we carry out a parameter study to find the right parameter β for the rat electrophysiology and the curves show the AP Φ (solid) evolution displayed alongside the recovery variable *r* (dashed) over time *t*. Another way to derive the same electrophysiological behavior for the rat is to adapt the conversion coefficient of time *k*_*t*_. The obtained parameter is then used to compute the normalized AP and the recovery variable, which are plotted over the normalized time $\bar{t}$ with initial values (ϕ = 0, *r* = 0) in Figure A1 (right). Roughly, the rat heart beats about four times faster than the human heart. To trigger the AP excitation, a stimulus *I* is required. As illustrated in Figure A1 (right), the AP then increases steeply in the depolarization phase. After reaching its maximum value of 1.0, the repolarization follows and it smoothly returns to its resting potential ϕ_*r*_ = 0. Apart from the non-pacemaker cells, there exist self-oscillating pacemaker cells, which can be modeled, e.g., by the FitzHugh-Nagumo model (Fitzhugh, 1961). In Figure A2 (left), for the choice of β = 0.55, the phase plane of the two variables ϕ, *r* is shown with the steady state of equilibrium at $\phi =\dot{\phi }=r=\dot{r}=0$ (red dot ). Trajectories of nine starting points of the model (black dot **•**) finally run into the stable equilibrium point. Four dashed nullclines, which are solutions of $\dot{\phi }={\bar{f}}^{\phi }\left(\phi ,r\right)=0$ (red-dashed lines) and $\dot{r}={\bar{f}}^{r}\left(\phi ,r\right)=0$ (green-dashed lines), pilot the solution trajectories of the model to the equilibrium point. This means that the parameters yield a stable model and the potential is only triggered by a stimulus. In Figure A2 (right), the correlation between AP Φ and active fiber tension *T*^*act*^ computed using (16) is illustrated. The two curves are similar since their relation is linear.

### A.4. Mechano-Electrical Feedback

For the electrical source term $F_{m}^{\Phi }$, its tangent terms with respect to AP and deformation are:

(A19) $$\begin{array}{l} \begin{array}{c} \partial_{\Phi }F_{m}^{\Phi }=-\vartheta G_{s}\left(\lambda -1\right),\;{\text{d}}_{\text{C}}F^{\Phi }=\frac{1}{2}\vartheta G_{s}\left(\Phi_{s}-\Phi \right)\lambda^{-1}f_{0}⊗f_{0}. \end{array} \end{array}$$

### A.5. Material Parameters

**Table A1:** Material parameters for simulation of rat cardiac muscles.

**Mechanical**	
Passive stress (TIC)	λ = 0.5 MPa, μ = 0.2 MPa, η = 0.1 MPa (Göktepe and Kuhl, 2009)
Active stress	*k*_*T*_ = 0.005 MPa mV^−1^, Φ_*r*_ = −80 mV
Passive stress (TII)	μ = 0.5 MPa, η = 0.2 MPa, κ=10^4^ kPa (model fit)
Active stress	*k*_*T*_ = 0.005 MPa mV^−1^, Φ_*r*_ = −80 mV
Passive stress (HO)	*a* = 0.144 kPa, *b* = 9.758 [-], *a*_*f*_ = 9.664 kPa, (model fit)
	*b*_*f*_ = 14.791 [-], *a*_*s*_ = 1.687 kPa, *b*_*s*_ = 7.336 [-],
	*a*_*fs*_ = 0.209 kPa, *b*_*fs*_ = 11.089 [-], κ=10^4^kPa
Active stress	*k*_*T*_ = 0.49 kPa mV^−1^, Φ_*r*_ = −80 mV, ν_*ff*_ = 1.0, ν_*ss*_ = 0.0 (Niederer et al., 2009)
Switch function	ϵ_0_ = 0.1 mV^−1^, ϵ_∞_ = 1.0 mV^−1^ (Göktepe and Kuhl, 2010)
	ξ = 1.0 mV^−1^, $\tilde{\Phi }$ = 0 mV
**Electrical**	
Conduction	*d*_*iso*_ = 0.1 mm^2^/mm^−1^, *d*_*ani*_ = 0.3 mm^2^/ms^−1^
Excitation	α = 0.01 [-], β = 0.55 [-], *c* = 8 [-] (parameter study)
	γ = 0.002 [-], μ_1_ = 0.2 [-], μ_2_ = 0.3 [-],
	*G*_*s*_ = 10 [-], ϕ_*s*_ = 0.6 [-]
Conversion	*k*_ϕ_ = 100 mV, δ_ϕ_ = 80 mV, *k*_*t*_ = 12.9 ms (Aliev and Panfilov, 1996)
